# Diverse Cell Death Mechanisms Are Simultaneously Activated in Macrophages Infected by Virulent *Mycobacterium tuberculosis*

**DOI:** 10.3390/pathogens11050492

**Published:** 2022-04-21

**Authors:** Lucero A. Ramon-Luing, Yessica Olvera, Julio Flores-Gonzalez, Yadira Palacios, Claudia Carranza, Yerany Aguilar-Duran, Marco Antonio Vargas, Neptali Gutierrez, Karen Medina-Quero, Leslie Chavez-Galan

**Affiliations:** 1 Laboratory of Integrative Immunology, Instituto Nacional de Enfermedades Respiratorias “Ismael Cosío Villegas”, Mexico City 14080, Mexico; ramonluing@yahoo.com.mx (L.A.R.-L.); jfloresg1707@alumno.ipn.mx (J.F.-G.); yadpal@gmail.com (Y.P.); haduran.yerany@gmail.com (Y.A.-D.); 2 Research Department, Military School of Graduate of Health, SEDENA, Mexico City 11200, Mexico; acisey999@gmail.com (Y.O.); labbiomol.emgs@udefa.edu.mx (M.A.V.); gutierrezdlc911@gmail.com (N.G.); labinmuno.emgs@udefa.edu.mx (K.M.-Q.); 3 Laboratory of Tuberculosis Immunobiology, Instituto Nacional de Enfermedades Respiratorias “Ismael Cosio Villegas”, Mexico City 14080, Mexico; carranza.salazar.claudia@gmail.com

**Keywords:** macrophages, tuberculosis, virulence, apoptosis, necroptosis, pyroptosis

## Abstract

Macrophages are necessary to eliminate pathogens. However, some pathogens have developed mechanisms to avoid the immune response. One of them is modulating the cell death mechanism to favor pathogen survival. In this study, we evaluated if virulent *Mycobacterium tuberculosis* (*M. tb*) can simultaneously activate more than one cell death mechanism. We infected human monocyte-derived macrophages (MDM) in vitro with avirulent (H37Ra) and virulent (H37Rv) strains, and then we measured molecules involved in apoptosis, necroptosis, and pyroptosis. Our data showed that H37Rv infection increased the BCL-2 transcript and protein, decreased the *BAX* transcript, and increased phosphorylated BCL-2 at the protein level. Moreover, H37Rv infection increased the expression of the molecules involved in the necroptotic pathway, such as ASK1, p-38, RIPK1, RIPK3, and caspase-8, while H37Ra increased caspase-8 and decreased *RIPK3* at the transcriptional level. In addition, *NLRP3* and *CASP1* expression was increased at low MOI in both strains, while IL-1β was independent of virulence but dependent on infection MOI, suggesting the activation of pyroptosis. These findings suggest that virulent *M. tb* inhibits the apoptosis mediated by BCL-2 family molecules but, at the same time, increases the expression of molecules involved in apoptosis, necroptosis, and pyroptosis at the transcriptional and protein levels, probably as a mechanism to avoid the immune response and guarantee its survival.

## 1. Introduction

Tuberculosis (TB) is an infectious disease caused by *Mycobacterium tuberculosis* (*M. tb*). In the most recent report of the World Health Organization (WHO), there were 10 million new cases of TB and a mortality of 1.3 million among human immunodeficiency virus-negative people, up from 1.2 million reported in 2019 [1]. It is noteworthy that this is the first time the WHO has reported an increase in TB mortality (1.3 million). 

The activation of immune cells during infection is necessary to eliminate pathogens [2]. Macrophages are one of the first cells activated in response to an infection; their precursor cells are circulating monocytes, which migrate into the tissue and are differentiated into resident macrophages [3]. There are several macrophage subpopulations, although the best described are the M1 or classically activated macrophages, characterized by secreting pro-inflammatory cytokines, and the M2 or alternatively activated macrophages, which deliver an anti-inflammatory cytokine profile [4]. In 2011, a new macrophage subpopulation that expresses the CD3/T cell receptor (TCR) complex (hereafter called CD3+ macrophages) was reported. These CD3+ macrophages have a pro-inflammatory function, and they secrete cytokines such as Tumor Necrosis Factor (TNF), Interferon-gamma (INF-γ), and Interleukin (IL)-6 via a CD3 dependent pathway [5,6]. Reports suggest that CD3+ macrophages are recruited at the infection site and play an essential role during mycobacterial infections. This has been observed both in granuloma from TB patients and in murine models of BCG infection [5,6,7]. It has recently been reported that although a short time (24 h) of infection with virulent *M. tb* is not enough to increase the frequency of CD3+ macrophages, it is sufficient to increase that of CD3+TCRαβ+ macrophages [8].

Macrophages activate defense mechanisms to limit the intracellular growth of *M. tb*; however, several reports have also demonstrated that *M. tb* activates mechanisms to prevent elimination and promote disease progression [9]. In this regard, reports indicated that virulent *M. tb* manipulates cell death pathways to survive, blocking apoptosis and activating other cell death pathways such as necrosis, which allows the spread of the bacteria [10,11]. 

The proteins of the B cell lymphoma 2 (BCL-2) family are involved in apoptosis regulation. They are classified into three groups according to their functions in apoptosis and the number of Bcl-2 homology (BH) domains they possess. Myeloid cell leukemia 1 (MCL-1) and BCL-2 proteins display anti-apoptotic functions, while BCL-2 associated X-apoptosis regulator (BAX) and BCL2 Antagonist/Killer 1 (BAK) proteins are pro-apoptotic regulators. In addition, pro-apoptotic BH3-only proteins promote mitochondrial outer membrane permeabilization (MOMP) [12]. Furthermore, BCL-2 proteins regulate the mitochondrial apoptosis pathway associated with the release of the pro-apoptotic mitochondrial protein SMAC/DIABLO (second mitochondria-derived activator of caspase/direct inhibitor of apoptosis-binding protein with low pI) [13].

Reports have indicated that *M. tb* induces other types of cell death to favor its survival. In some of these types, cytokines play an essential role in activating the cell death of infected macrophages. For instance, the pathway mediated by Interleukin 1 beta (IL-1β) triggers a cell death mechanism called pyroptosis [14]. Indeed, M tb infection causes caspase-1/NLRP3/gasdermin D-mediated pyroptosis in human monocytes and macrophages, in which plasma membrane damage activates NLRP3-dependent IL-1β release and pyroptosis [15]. *M. tb* also induces necrotic-like cell death, a pathway that promotes the spread of bacilli [16]. Molecules such as receptor-interacting serine/threonine-protein kinase 1 and kinase 3 (RIPK1 and RIPK3) are involved in this type of necrosis, also referred to as necroptosis [17,18,19]. Furthermore, it has been shown that the hydrolysis of nicotinamide adenine dinucleotide (NAD+) by the tuberculosis necrotizing toxin secreted by *M. tb* activates RIPK3 and MLKL, the main mediators of necroptosis [20]. 

The specific type of cell death process activated in *M. tb*-infected macrophages is fundamental for the progression of infection. However, it is still unclear if *M. tb* virulent strains simultaneously activate apoptosis, pyroptosis, and necroptosis. Therefore, in this study, we evaluated some key intracellular molecules involved in these pathways, both at the transcriptional and protein levels, to identify how virulent *M. tb* could regulate cell death by favoring one or more specific pathways to ensure its survival.

## 2. Results

### 2.1. M. tb Increases BCL-2 and Decreases BAX at the Transcriptional Level, Independently of Its Virulence

We investigated whether *M. tb* virulence influences the expression of members of the BCL-2 family. First, we quantified the expression of two anti-apoptotic genes, *BCL-2* and *MCL-1*, and two pro-apoptotic genes, *BAX* and *DIABLO*, at the transcriptional level. 

**Figure 1:**
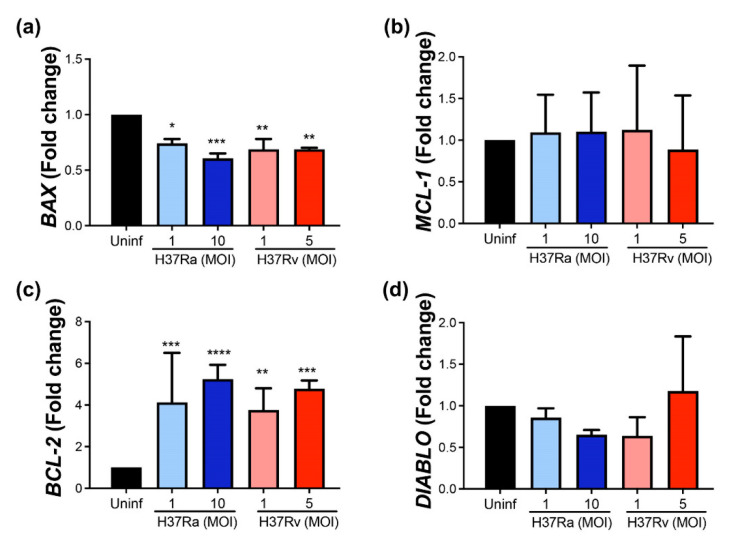
*M. tb* virulent and avirulent strains increase the gene expression of the anti-apoptotic *BCL-2* and decrease the pro-apoptotic *BAX*. Human MDMs were obtained after seven days in culture. 2 × 10^6^ MDMs were infected at MOI 1 and MOI 10 with an avirulent (H37Ra) and at MOI 1 and MOI 5 with a virulent (H37Rv) strain of *M. tb*; 2 × 10^6^ MDMs were not infected as a control (Uninf). At 24 h postinfection, cells were recovered and prepared for qPCR. Relative expression of *BCL-2* (**a**), *MCL-1* (**b**), *BAX* (**c**), and *DIABLO* (**d**) genes was assessed using qPCR. Bar graphs show the mean ± SD from four independent experiments (*n* = 4 donors). Statistical analysis was performed using Kruskal–Wallis analysis, followed by Dunn’s post hoc test. * *p* < 0.05, ** *p* < 0.01, *** *p* < 0.001, **** *p* < 0.0001.

Our data showed that *BCL-2* gene expression increased four-fold in response to *M. tb* infection and was induced by both H37Ra and H37Rv strains compared to uninfected MDM. Similar results were obtained using high and low MOI (Figure 1a). In contrast, *MCL-1* gene expression was not affected (Figure 1b). Contrary to what we observed for the anti-apoptotic genes, both *M. tb* strains decreased *BAX* expression by around 30% in MDM (Figure 1c) independently of the MOI, while *DIABLO* expression was not modified (Figure 1d).

These results suggest that *M. tb* increases the expression of anti-apoptotic *BCL-2* and decreases the pro-apoptotic *BAX* at the transcriptional level, independently of virulence.

### 2.2. Virulent M. tb Increases p-BCL-2 

**Figure 2:**
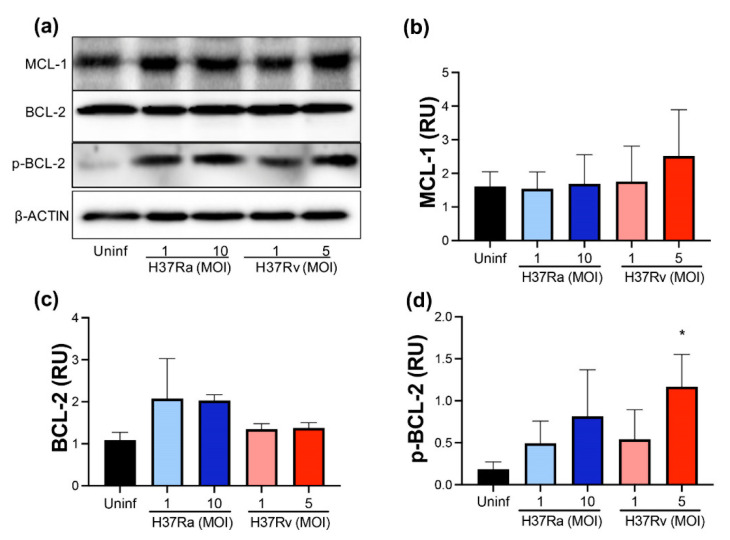
*M. tb* virulence induces phosphorylation of BCL-2. Human MDMs were obtained after seven days in culture. 2 × 10^6^ MDMs were infected at MOI 1 and MOI 10 with an avirulent (H37Ra) and MOI 1 and MOI 5 with a virulent (H37Rv) strain of *M. tb*; 2 × 10^6^ MDMs were not infected as a control (Uninf). At 24 h postinfection, cells were recovered and prepared for Western Blot. Representative Western blot of MCL-1, BCL-2, p-BCL-2, and β-ACTIN (**a**). Band densities of MCL-1 (**b**), BCL-2 (**c**), and p-BCL-2 (**d**) were normalized against β-ACTIN and quantified by densitometry analysis with the ImageJ software. Results are shown in relative units (RU) of concentration. The bar graphs show the mean ± SD from two independent experiments (MCL-1 and BCL-2, *n* = 2 donors and two technical replicates; p-BCL-2, *n* = 3 donors and two technical replicates). Statistical analysis was performed using Kruskal–Wallis analysis, followed by Dunn’s post hoc test. * *p* < 0.05.

Subsequently, we measured the anti-apoptotic molecules MCL-1 and BCL-2 at the protein level to confirm their expression at the transcriptional level. The anti-apoptotic proteins MCL-1, BCL-2, and p-BCL-2 were quantified using Western blot (Figure 2a). In concordance with the transcription level, the MCL-1 protein level was not modified after infection with either an avirulent or a virulent *M. tb* strain (Figure 2b). Unexpectedly, we did not observe a difference in the BCL-2 protein level in infected compared to uninfected MDMs (Figure 2c). The discordance in BCL-2 expression between transcriptional and protein levels could result from the activation of BCL-2 to mediate an anti-apoptotic function. Therefore, the BCL-2 phosphorylated form (p-BCL-2) was evaluated to confirm this hypothesis. Results showed that both *M. tb* strains, only with high MOI (10 and 5, for avirulent and virulent strains, respectively), increased the p-BCL-2 level. However, H37Rv infection with MOI 5 induced a significant increase compared to uninfected MDMs (1.4 RU vs. 0.1 RU) (Figure 2d). These findings suggest that H37Rv infection activates anti-apoptotic BCL-2-dependent mechanisms in MDMs.

### 2.3. M. tb Infection Does Not Modify Pro-Apoptotic Proteins

**Figure 3:**
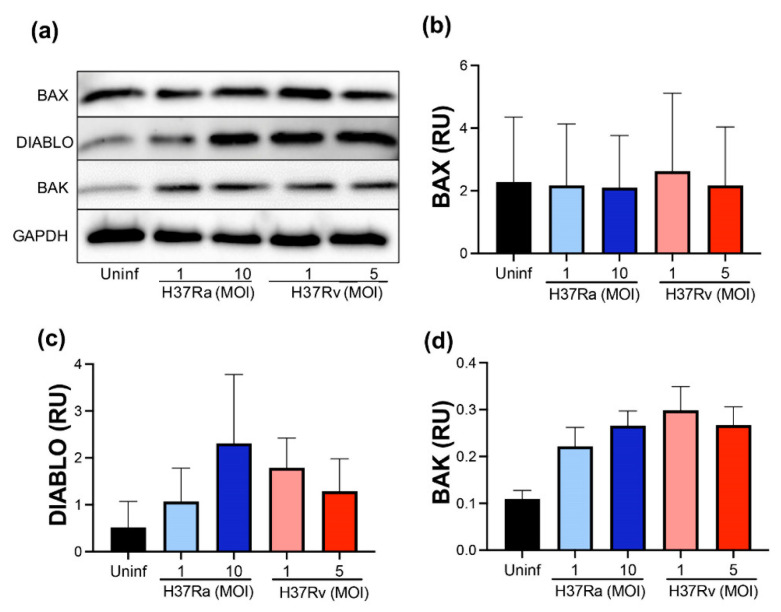
*M. tb* infection does not modify pro-apoptotic proteins. Human MDMs were obtained after seven days in culture. 2 × 10^6^ MDMs were infected at MOI 1 and MOI 10 with an avirulent (H37Ra) and MOI 1 and MOI 5 with a virulent (H37Rv) strain of *M. tb*; 2 × 10^6^ MDMs were not infected as a control (Uninf). At 24 h postinfection, cells were recovered and prepared for Western Blot. Representative Western blot of BAX, DIABLO, BAK, and GAPDH (**a**). Band densities of BAX (**b**), DIABLO (**c**), and BAK (**d**) were normalized against and quantified by densitometry analysis with the ImageJ software. Results are shown in relative units (RU) of concentration. The bar graphs show the mean ± SD from two independent experiments (*n* = 2 donors and two technical replicates). Statistical analysis was performed using Kruskal–Wallis analysis, followed by Dunn’s post hoc test.

For further confirmation of the pro-apoptotic molecules at the transcriptional level, we evaluated BAX, DIABLO, and BAK at the protein level using Western blot (Figure 3a). We did not detect differences in BAX and DIABLO at the protein level comparing infected with uninfected MDM (Figure 3b,c, respectively). In addition, BAK is a pro-apoptotic protein that, together with BAX, induces the mitochondrial membrane pore to allow cytochrome C to exit; thus, the evaluation of BAK was included to discard that an excess of BAK masks the BAX level. However, we did not observe any significant difference at the protein level of BAK (Figure 3d). Data suggest that the pro-apoptotic proteins evaluated are not increased either with avirulent or virulent *M. tb*.

### 2.4. High MOI of Virulent M. tb Increases ASK1 and p-P38 Expression

**Figure 4:**
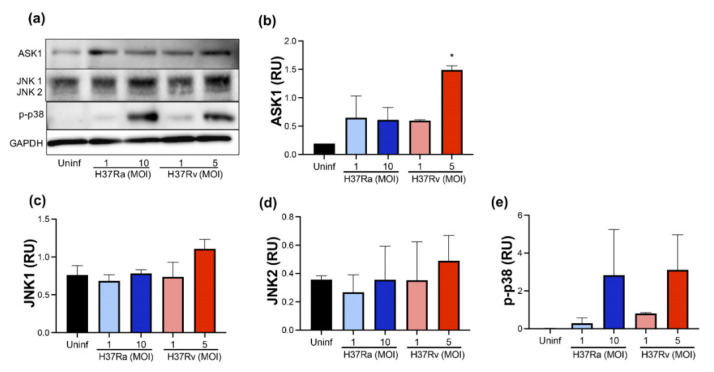
*M. tb* virulence induces ASK1 expression. Human MDMs were obtained after seven days in culture. 2 × 10^6^ MDMs were infected at MOI 1 and MOI 10 with an avirulent (H37Ra) and MOI 1 and MOI 5 with a virulent (H37Rv) strain of *M. tb*; 2 × 10^6^ MDMs were not infected as a control (Uninf). At 24 h postinfection, cells were recovered and prepared for Western Blot. Representative Western blot of ASK1, JNK1, JNK2, p-p38, and GAPDH (**a**). Band densities of ASK1 (**b**), JNK1 (**c**), JNK2 (**d**), and p-p38 (**e**) were normalized against GAPDH and quantified by densitometry analysis with the ImageJ software. Results are shown in relative units (RU) of concentration. The bar graphs show the mean ± SD from two independent experiments (*n* = 2 donors and two technical replicates). Statistical analysis was performed using Kruskal–Wallis analysis, followed by Dunn’s post hoc test. * *p* < 0.05.

The MAP kinases family plays several roles in the cell; JNK1/2, ASK1, and p38 are kinases that, under certain conditions, participate in cell death pathways [21,22]. Therefore, we evaluated the presence of ASK1, JNK1/2, and p-p38 at the protein level using Western blot (Figure 4a).

Our data showed that H37Rv at MOI 5 increased ASK1 compared with uninfected MDM (1.5 RU versus 0.2 RU, respectively) (Figure 4b). Furthermore, JNK1 and JNK2 were not affected by the infection of either the H37Ra or the H37Rv strain (Figure 4c,d, respectively). We observed that a high MOI of *M. tb* increased the p-p38 level from an undetectable level in uninfected MDM to 3 RU with both H37Ra (MOI 10) and H37Rv (MOI 5) infection (Figure 4e). However, this increase was not statistically significant. These results showed that the ASK1/p38 axis increases during infection by a virulent *M. tb* strain, suggesting an association with alternative cell death pathways, such as apoptosis or necroptosis.

### 2.5. A High MOI of H37Ra Decreases RIPK3 Expression at the Transcriptional Level, Whereas a High MOI of Either M. tb Strain Increases the Phosphorylated RIPK1 Protein Level

Necroptosis is another type of cell death where RIPK1 and RIPK3 play an essential role [18,19]. Therefore, we analyzed if the virulence of *M. tb* affects the expression of these molecules at the transcriptional and protein levels. 

**Figure 5:**
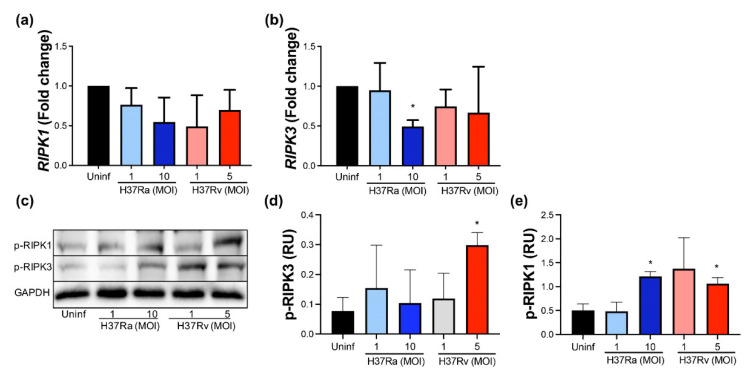
Increased MOI from the non-virulent strain of *M. tb* inhibits *RIPK3* gene expression, while the virulent strain favors RIPK1 and RIPK3 expression at the protein level. Human MDMs were obtained after seven days in culture. 2 × 10^6^ MDMs were infected at MOI 1 and MOI 10 with an avirulent (H37Ra) and at MOI 1 y MOI 5 with a virulent (H37Rv) strain of *M. tb* 24 h; 2 × 10^6^ MDMs were not infected as a control (Uninf). After postinfection, cells were recovered and prepared for qPCR or Western Blot. Relative expression of RIPK1 (**a**) and RIPK3 (**b**) genes was assessed by qPCR. Bar graphs show the mean ± SD from four independent experiments (*n* = 4 donors). Representative Western blot of p-RIPK1, p-RIPK3, and GAPDH (**c**). Band densities of p-RIPK1 (**d**) and p-RIPK3 (**e**) were normalized against GAPDH and quantified by densitometry analysis with the ImageJ software. Results are shown in relative units (RU) of concentration using ImageJ software. The bar graphs show the mean ± SD from two independent experiments (*n* = 2 to immunoblot, and *n* = 4 to qPCR were performed with two technical replicates). Statistical analysis was performed using Kruskal–Wallis analysis, followed by Dunn’s post hoc test. * *p* < 0.05.

Our data showed that neither avirulent nor virulent *M. tb* affect the expression of the *RIPK1* gene (Figure 5a), but the *RIPK3* gene expression decreased to near 50% when MDMs were infected with H37Ra MOI 10 compared to uninfected MDM (Figure 5b). Subsequently, these proteins were measured using Western blot (Figure 5c). Data showed that phosphorylated RIPK1 increased two-fold in the infected MDMs with a high MOI of either *M. tb* strain, while phosphorylated RIPK3 increased three-fold with a high MOI of H37Rv. These data suggest that the virulence of *M. tb* influences RIPK1 and RIPK3.

### 2.6. Virulent M. tb Favors an Increase of Processed Caspase-8

**Figure 6:**
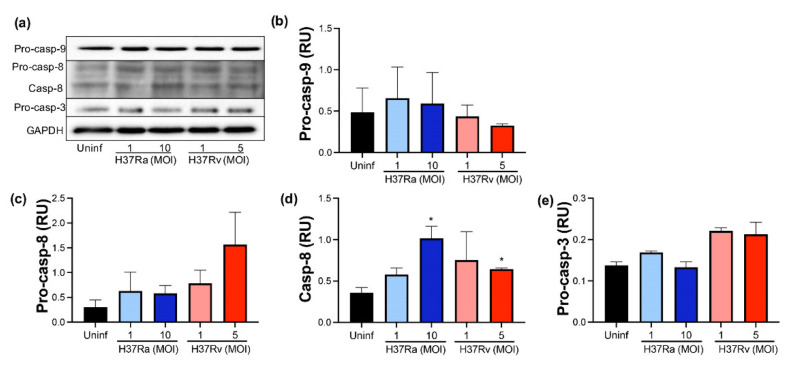
The presence of virulent *M. tuberculosis* induces the expression and activation of caspase-8. Human MDMs were obtained after seven days in culture. 2 × 10^6^ MDMs were infected at MOI 1 and MOI 10 with an avirulent (H37Ra) and MOI 1 and MOI 5 with a virulent (H37Rv) strain of *M. tb*; 2 × 10^6^ MDMs were not infected as a control (Uninf). At 24 h postinfection, cells were recovered and prepared for Western Blot. Representative Western blot of Pro-caspase-9, Pro-caspase-8, Caspase-8, Pro-caspase-3, and GAPDH (**a**). Band densities of Pro-caspase-9 (**b**), Pro-caspase-8 (**c**), Caspase-8 (**d**), and Pro-caspase-3 (**e**) were normalized against GAPDH and quantified by densitometry analysis with the ImageJ software. Results are shown in relative units (RU) of concentration. The bar graphs show the mean ± SD from two independent experiments (*n* = 2 donors and two technical replicates). Statistical analysis was performed using Kruskal–Wallis analysis, followed by Dunn’s post hoc test. * *p* < 0.05.

The caspase–cascade system is necessary to amplify the intracellular apoptotic signals. Thus, we evaluated both zymogen (pro-caspase) and active forms of two initiators, caspases (caspase-8 and caspase-9) and one effector caspase (caspase-3), using Western blot (Figure 6a). 

Our data showed that pro-caspase-9 was not increased by *M. tb* infection, and we did not detect processed caspase-9 (Figure 6b). Regarding caspase-8, apparently, H37Rv MOI 5 increased the pro-caspase-8 level compared to uninfected MDM (1.5 RU versus 0.4 RU, respectively), but it was not statistically significant (Figure 6c); moreover, the *CASP8* gene was not differentially expressed depending on *M. tb* virulence (data not shown). Furthermore, the processed caspase-8 level increased up to 1 RU after *M. tb* infection with a high MOI of H37Ra, while with low and high MOI of H37Rv, it increased up to 0.9 and 0.7, respectively (Figure 6d). Although this increase does not represent a substantial change compared to uninfected conditions, results suggest that *M. tb* could favor the activating of caspase-8. Finally, we did not find the processed caspase-3 in any infection condition (Figure 6e). 

### 2.7. Virulent M. tb Increases the Expression of NLRP3 and IL-1β, Molecules Involved in Pyroptosis

Pyroptosis is a type of cell death in which NLRP3 and IL-1β play an essential role [14,23]. Therefore, *NLRP3* and *IL-1β* gene expression was evaluated, and IL-1β soluble protein was quantified. 

**Figure 7:**
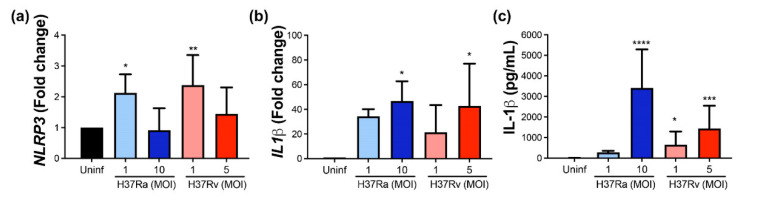
The virulence of *M. tb* positively modifies the expression and delivery of IL-1β. Human MDMs were obtained after seven days in culture. 2 × 10^6^ MDMs were infected at MOI 1 and MOI 10 with an avirulent (H37Ra) and at MOI 1 y MOI 5 with a virulent (H37Rv) strain of *M. tb* 24 h postinfection cells were recovered and prepared for qPCR, and supernatant for ELISA. Relative gene expression of *NLRP3* (**a**) and *IL-1β* (**b**) genes was assessed by qPCR and protein level of IL-1β by ELISA (**c**). Bar graphs show the mean ± SD from four or six independent experiments performed with two technical replicates. Statistical analysis was performed using Kruskal–Wallis analysis, followed by Dunn’s post hoc test. * *p* < 0.05, ** *p* < 0.01, *** *p* < 0.001, **** *p* < 0.0001.

Data showed that *NLRP3* gene expression was increased after *M. tb* infection with both strains at a low MOI (Figure 7a), while *IL-1β* gene expression was increased at a high MOI of *M. tb* independently of the virulence (Figure 7b). Finally, a high MOI of H37Ra increased the IL-1β soluble protein (up to 3800 pg/mL) compared with the soluble protein delivered by uninfected MDM (undetectable level). Furthermore, the virulent *M. tb* H37Rv strain increased the level of this pro-inflammatory cytokine at low and high MOI (800 and 1800 pg/mL, respectively). This result confirms that virulent *M. tb* also modifies molecules involved in the activation of pyroptosis.

### 2.8. Virulent M. tb Favors Further Necrosis and the CASP1 Expression at the Transcriptional Level

**Figure 8:**
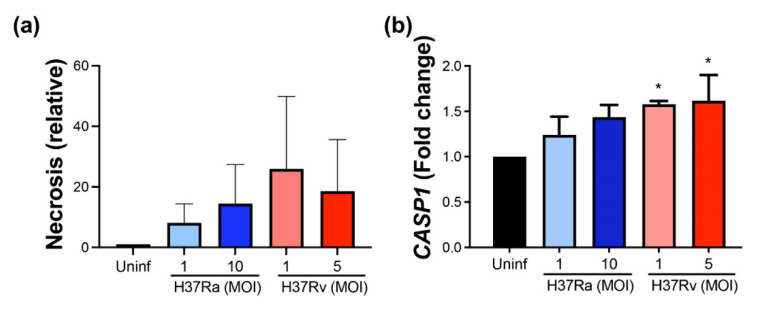
*M. tb* infection induces necrosis, and virulent *M. tb* increases further *CASP1* expression. MDMs were obtained after seven days in culture. 2 × 10^6^ MDMs were infected at MOI 1 and MOI 10 with an avirulent (H37Ra) and at MOI 1 y MOI 5 with a virulent (H37Rv) strain of *M. tb* 24 h postinfection cells were recovered and prepared for qPCR, and supernatant for ELISA. Histone-associated DNA fragments (mono- and oligonucleosomes) were quantified in supernatants. Results are shown as a relative increase compared with uninfected conditions. Necrosis was assessed by cell death detection ELISA (**a**) and relative gene expression of *CASP1* gene by qPCR (**b**). Bar graphs show the mean ± SD from three donors performed with two technical replicates. Statistical analysis was performed using Kruskal–Wallis analysis, followed by Dunn’s post hoc test. * *p* < 0.05.

For further confirmation of the cell death process as a global mechanism induced by *M. tb* infection, we evaluated the release of histone-complexed DNA fragments as an indirect measure of necrotic cells. Results showed that relative necrosis is generally increased by *M. tb* infection, and to a larger extent by virulent Mt.tb, although it is not significant statistically (Figure 8a). Furthermore, *CASP1* expression was considered to be indicative of pyroptosis triggered by mycobacteria. We found increased *CASP1* at the transcriptional level with virulent *M. tb* at low and high MOI (Figure 8b). These data confirm that *M. tb* infection induces cell death, and virulence favors pyroptosis.

## 3. Discussion

Macrophages play a critical role against *M. tb* infection. *M. tb* has developed diverse mechanisms to evade the immune response and promote its survival within the cell [24]. The virulence of *M. tb* modulates diverse macrophage functions. Recent findings show that H37Rv infection increases the migration frequency of pro-inflammatory CD3+TCRαβ+ macrophages [8]. It is probable that mycobacterial virulence allows the arrival of specific macrophages subpopulations. Although cell death should be a process regulated by host cells to eliminate mycobacteria, *M. tb* seems to be able to control cell death to escape the immune response [25,26]; the understanding of the immune, autophagic, and cell-death responses during TB is not yet complete; thus, some questions remain to be addressed. Interestingly, mitochondrial damage has been reported in monocytes of TB patients, which could render these cells more susceptible to cell death [27]. In consonance, *M. tb* infection in mice has been shown to induce a dysregulation in the mitochondrial membrane potential in myeloid progenitors, to induce an unanticipated immune evasion in bone marrow and to control the intrinsic antimicrobial capacity of innate immunity, a process suggested as innate immunity training in some infections [28,29]. 

Thus, although it is well established that virulent *M. tb* inhibits apoptosis and activates necrosis to evade the immune response, there is controversy about how these mechanisms are modulated. For instance, while some studies suggest that the host uses apoptosis as a defense mechanism against *M. tb*, others indicate that virulent *M. tb* strains use apoptosis as a mechanism for colonization [30,31]. Regarding necrosis, it has been clearly associated with enhanced bacterial replication, promoting transmission and active disease [32,33,34]. Although, at present, it is not clear if other cell death mechanisms are activated simultaneously by virulent *M. tb*, it could be helpful to know how these mechanisms, such as pyroptosis and necroptosis, have been regulated to favor bacillus spread. In this study, we evaluated if the virulent *M. tb* could activate these cell death pathways and if this is simultaneous or one by one.

Bacterial load and virulence play a central role in the outcome of the *M. tb* infection; therefore, identifying the molecular mechanisms involved in the switch of cell death pathways could be relevant for the control of *M. tb*. Furthermore, although apoptosis has been broadly described, there is no clear understanding of the effect of *M. tb* infection on cellular processes, such as intracellular membrane trafficking and integrity, pyroptosis, necroptosis, ferroptosis, NETosis, autophagy, and inflammasome activation [25].

The canonical models of cell death proposed for the regulation of molecules of the BCL-2 family stipulate that BCL-2 anti-apoptotic proteins block the activation of the effectors pro-apoptotic BAX and BAK. In cell activation, the last two proteins trigger MOMP and the subsequent release of apoptogenic factors, such as cytochrome-c, which is involved in mitochondrial cell death [12,35]. However, reports indicate that *M. tb* regulates diverse mechanisms inside macrophages to ensure their survival. For instance, the avirulent H37Ra increases cyclooxygenase 2 (COX2), inducing prostaglandin E2 (PGE2), which protects the mitochondrial inner membrane, and inhibits lipoxin A4 (LXA4), leading to apoptosis activation [36,37]. In contrast, virulent H37Rv induces necrosis through the increase in LXA4 and reduction in PGE2 [38]. Furthermore, it was shown that mycobacterial infection differentially regulated pro- and anti-apoptotic molecules of the BCL-2 family, but we did not observe an increase in MCL-1, as previously reported [39]. This discrepancy could be explained by a different cell origin. 

We observed that H37Rv increases the *BCL-2* gene expression and a high level of p-BCL-2; this suggests that virulent *M. tb* activates an anti-apoptotic pathway, which is BCL-2 dependent. MCL-1 and BCL-2 have anti-apoptotic functions. MCL-1 inhibits cytochrome-c release and subsequent mitochondrial membrane permeabilization. Although it has been suggested that MCL-1 can be found in both acute and chronic models of *M. tb* infection, the MCL-1 expression increases mainly after 16 weeks of infection, accompanied by a change in the macrophage phenotype towards M2, characterized by an anti-inflammatory profile [40]. In our study, we found an infection time of 24 h, and the cytokine profile suggests a pro-inflammatory microenvironment; the MCL-1 level probably requires a more prolonged time of infection to be modulated and could be associated with the M2 phenotype. Like MCL-1, the pro-apoptotic DIABLO did not show any change, which is probably because BCL-2 plays a role in regulating its level; in this regard, it was recently reported that the DIABLO efflux is inhibited in BCL-2-overexpressing cells [41]. 

One of the limitations of this study is that we did not conduct a phenotypical characterization to differentiate the frequency of M1 or M2 macrophages. However, a high level of IL-1β also indicates a pro-inflammatory profile. Moreover, using this in vitro infection model, we recently reported an increase in the frequency of CD3+TCR+ macrophages [8], supporting the hypothesis that this model favors a pro-inflammatory profile.

In agreement with the increased level of p-BCL-2 observed as a consequence of H37Rv infection, we did not observe processed caspases-9 and -3, suggesting a suppression or delay of the mitochondrial apoptotic pathway under the evaluated conditions. However, the level of processed caspase-8 increased; here, it is important to note that H37Rv also increased the IL-1β level. In this regard, it has been reported that bacteria-infected macrophages activate a non-canonical pathway of the inflammasome in which caspase-8 and IL-1β can up-regulate their levels via a feedback mechanism [42,43]. Therefore, we suggest that the presence of caspase-8 is related to other mechanisms of cell death, such as pyroptosis; moreover, the increased level of RIPK1 and RIPK3 induced by H37Rv could be associated with the necroptosis mechanism, as it was previously reported that the caspase-8/RIPK/IL-1b axis is a key regulator of macrophage cell death [44].

Several reports have demonstrated that virulent *M. tb* can evade the immune response by modulating cell death mechanisms. In addition, in this study, we present evidence supporting the simultaneous activation of several cell death pathways by virulent *M. tb*. Other evidence that supports our data is the increased level of ASK1 protein; this protein has been associated with apoptosis. These reports also indicated that, in the context of mycobacterial infection, ASK1 could participate in cell death via necrosis at a high MOI [45]. Furthermore, the ASK1-mediated activation of JNK and p38 MAPKs in various stress conditions leads to cell death, inflammation, and fibrosis [46]. Finally, although there is a crosstalk between apoptosis and necroptosis facilitated by caspase-8 as a negative regulator of RIPK-1 during endoplasmic reticulum (ER) stress [47], the role of these enzymes in the crosstalk between apoptosis and necroptosis remains unclear. We know, though, that in some cases, caspase-8 behaves as a key switch regulator that establishes which cell death pathway will be activated [48].

Ferroptosis is another type of nonapoptotic cell death that is iron dependent and characterized by lipid peroxide accumulation. Although it is involved in physiological cellular processes, ferroptosis has also been associated with several diseases, including *M. tb* infection [49]. Iron plays an essential role in bacterial survival, but it is also necessary to produce ROSs, which have antibacterial effects [50]. Thus, ferroptosis depends on an excess of iron and lipid peroxidation under conditions of suppressed glutathione peroxidase-4 (Gpx4) [51]. The transcription factor nuclear factor erythroid 2-related factor 2 (NFR2) is an essential regulator of anti-ferroptotic genes, and some studies suggest that increased nitric oxide availability can induce their expression [52,53].

Evidence shows that ferroptosis is dependent on lipid peroxidation, glutathione (GSH) oxidation, and Gpx4 levels [54]. In this regard, a recent report suggests that therapy to reduce iron accumulation protects the host against mycobacterial infection [55]. Although we did not explore ferroptosis in this study, the observation mentioned above gives rise to an important question that should be answered in future studies since there is evidence suggesting a potential therapeutic role in active TB from blocking ferroptosis.

**Figure 9:**
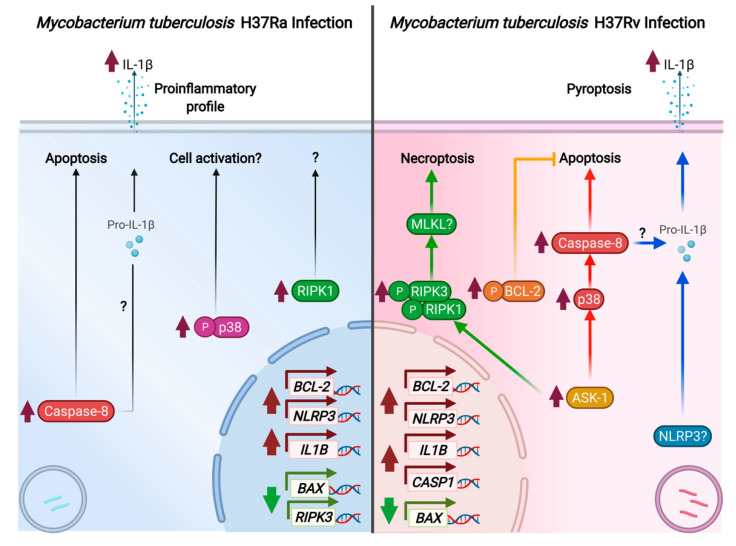
Representative scheme of death cell mechanisms induced by the *M. tuberculosis* infection.

In summary, our data showed that macrophages infected with the avirulent H37Ra strain exhibit increased caspase-8 and p-p38 levels and a pro-inflammatory microenvironment characterized by a high IL-1β level (Figure 9 left). On the contrary, infection with virulent H37Rv strain activates diverse cell death pathways, and this could be a strategy used by *M. tb* to warrant its survival. Herein, we found that virulent *M. tb* also activates multiple cell death mechanisms in the infected macrophage. In Figure 9 (right), our data show that H37Rv avoids BAX/BAK-mediated apoptosis, apparently via an increased level of p-BCL-2 (yellow line). Furthermore, ASK-1 kinase increases at the same time and can bind with diverse molecules and mediate diverse functions. In this way, while the ASK-1/p-p38 axis could facilitate the activation of caspase-8 and induce apoptosis (line red), ASK-1 could also activate the RIPK complex and mediate necroptosis (green line). Finally, we also observed an increase in the *NLRP3* and *CASP1* genes, together with the increased IL-1β both at transcriptional and protein levels; it is not surprising that it could mediate pyroptosis (blue line). In this process, IL-8 also could mediate IL-1β activation. In this study, we based our assessment of necrosis on the rupture of the plasma membrane, which is an event where several cell death pathways converge (even the apoptosis extrinsic pathway), leading to the release of cell components as fragmented DNA, and whose validity as a general marker of cell death has been proposed in previous reports [56]. Together, our results show that *M. tb* infection induces cell death, and the necrosis observed could be the result of both necroptosis and pyroptosis. It is noteworthy that pyroptosis, specifically, could be favored by *M. tb* virulence. 

## 4. Materials and Methods

### 4.1. Ethical Approval

The study was given ethical approval by the Institutional Review Board (IRB# B04-20) of the Instituto Nacional de Enfermedades Respiratorias Ismael Cosío Villegas (INER). This study was conducted according to the principles of the Declaration of Helsinki. Written informed consent for participation was not required for this study in accordance with the national legislation and institutional requirements.

### 4.2. Enrichment of CD14+ Cells and Generation of Monocyte-Derived Macrophages (MDM)

Peripheral blood mononuclear cells (PBMCs) were isolated from buffy coats of healthy donors attending the blood bank at the INER, using the standard LymphoprepTM (Accurate Chemical-Scientific, Westbury, NY, USA) gradient centrifugation. For the enrichment of monocytes, we used a method previously reported [8]. Briefly, PBMCs were cultured for 2 h in a Corning^®^ 100 mm TC-treated culture dish (Corning, New York, NY, USA), allowing them to adhere. After removing non-adherent cells, only adhered cells were recovered using a cell scraper, and posteriorly CD14+ cells were obtained via a positive selection using magnetic microbeads coated with an anti-CD14 monoclonal antibody (Miltenyi Biotech, Bergisch Gladbach, Germany). The purity of the CD14+ cell fraction was analyzed by flow cytometry with anti-human CD14, CD2, and CD19 monoclonal antibodies (mAbs) provided by BioLegend (San Diego, CA, USA). The enrichment efficiency of the CD14+ cell fraction was >96%. More information about Abs is shown in Table A1.

For MDMs generation, CD14+ cells were cultured in 6-well plates at a density of 2 × 10^6^ cells/well (Costar, Ontario, Canada) with RPMI-1640 culture medium (GIBCO, Grand Island, NY, USA) supplemented with 2 mM L-glutamine (GIBCO), 100 μg/mL streptomycin, 100 IU/mL penicillin, and 10% fetal bovine serum (FBS, GIBCO) for 7 days at 37 °C, under an atmosphere of 5% CO2. After culture, MDMs were characterized using immunophenotyping based on the expression of differentiation molecules, as previously reported [6].

### 4.3. In Vitro Infection Assays

To Mtb-infected MDM assays, *M. tb* laboratory strain stocks (H37Rv, ATCC25618; H37Ra ATCC25177, Manassas, VA, USA) were added, which were prepared and cultured as previously reported [57]. After 21 days of culture, the mycobacterial stock solution was harvested to prepare aliquots and then stored at −70 °C until use for in vitro infection assays. 

Infection assays were performed as we previously reported [8]. First, a bacteria suspension was prepared for macrophage infection, and an aliquot of bacteria was thawed and centrifuged at 6000× *g* for 5 min. Next, the obtained bacterial pellet was resuspended in RPMI medium supplemented with 10% human serum; after that, mycobacteria were declumped, and single-cell suspensions of bacteria were used for the infection of MDMs. 

MDMs (2 × 10^6^/mL) were infected with H37Ra at a multiplicity of infection (MOI) of 1 and 10 (1 cell per 1 or 10 bacilli, respectively) and with H37Rv at MOI 1 and 5 (1 cell per 1 or 5 bacilli, respectively). The infected MDMs were incubated at 37 °C for 2 h, and then nonphagocytosed bacteria were eliminated by washing, and MDMs were further incubated for 24 h at 37 °C.

### 4.4. Analysis of Gene Expression by Quantitative Real-Time PCR

After in vitro infection assays, 2 ×10^6^ infected MDMs from each condition were recovered, suspended with DNA/RNA Shield solution (Zymo Research, Irvine, CA, USA), and stored at −70 °C until use for RNA purification. First, total RNA was purified using the RNeasy Micro Kit (Qiagen, Hilden, Germany) according to the manufacturer’s instructions, and genomic DNA was eliminated with the RNA-Free DNAse Set (Qiagen). RNA was quantified using the Qubit™ assay kit and the Qubit 2.0 Fluorometer (Life Technologies, Waltham, MA, USA). Next, cDNA was synthesized from 200 ng of total RNA using the High-Capacity cDNA Reverse Transcription Kit (Applied Biosystems, Waltham, MA, USA) in a reaction volume of 20 μL, following the manufacturer’s guidelines. Finally, gene expression was evaluated with quantitative real-time PCR (qPCR) using TaqMan probes for the target genes, *BCL-2* (Hs04986394_s1), *MCL-1* (Hs06626047_g1), *BAX* (Hs00180269_m1), *DIABLO* (Hs00219876_m1), *IL-1B* (Hs01555410_m1), *RIPK1* (Hs01041869_m1), *RIPK3* (Hs00179132_m1), *NLRP3* (Hs00918082_m1), and *CASP1* (Hs00354836_m1); *18S* (18S ribosomal RNA gene) (Hs03928990_g1) and *ACTB* (β-actin) (Hs01060665_g1) were used as endogenous controls. For qPCR, single reactions were prepared using the Maxima Probe/ROX qPCR Master Mix (Thermo Fisher Scientific, Waltham, USA) and performed per duplicate under the following thermal conditions: 95 °C for 10 min, followed by 40 cycles of 60 °C for 1 min and 95 °C for 15 s, in the Step One Plus Real-Time PCR System (Applied Biosystems). 

Relative gene expression was determined using the ΔΔCT method to calculate the n-fold change for each target gene under each experimental condition. Results were normalized to the endogenous controls, ACTB and 18S genes, and relative to the reference group, uninfected MDMs (RQ = 2^−ΔΔCT^ = 1).

### 4.5. Western Blot Assay

*M. tb*-infected cells were lysed in Solulyse-M, Mammalian protein extraction reagent- Tris-sucrose buffer, pH 7.4 (Genlantis, San Diego, CA, USA), containing a protease inhibitor cocktail (Sigma-Aldrich, Fluka, MO, USA). Then, 50 μg of protein from whole-cell lysates was separated in 1-SDS-PAGE on 10% or 12% polyacrylamide gels. Then, proteins were transferred to a polyvinylidene difluoride (PVDF) membrane (Bio-Rad, Hercules, CA, USA). For Western blot, membranes were incubated with antibodies (Abs) to BCL-2, phosphorylated BCL-2 [(p-BCL-2, (Ser70)], MCL-1, BAX, BAK, DIABLO, ASK1, JNK1/2, phosphorylated p38 MAP Kinase [(p-p38, (T180/Y182)], phosphorylated RIPK1 [(p-RIPK1 (Ser166)], phosphorylated RIPK3 [(p-RIPK3 (Ser232)], caspase-9 (casp-9), caspase-8 (casp-8), and caspase-3 (casp-3). All Abs were used at a dilution of 1:1000 and incubated overnight at 4 °C. Abs anti-rabbit, anti-mouse, and anti-sheep conjugated to horseradish peroxidase (HRP) were used as a secondary antibody. In addition, GAPDH and β-actin were used as loading controls. More information about Abs is shown in Table A1.

Protein bands were visualized with the enhanced chemiluminescence reagent (Thermo Scientific, Pierce Biotech., Rockford, IL, USA) using an Imaging System from Bio-Rad (ChemiDocTM XRS+ System). Band densities were analyzed by densitometry using online ImageJ 1.39c software provided by the NIH and shown as relative units (RU), as previously reported [16]. 

### 4.6. ELISA Sandwich Assays

Supernatants recovered after infection assays were stored at −70 ◦C until analysis. The soluble level of IL-1β was quantified with the human IL-1β ELISA MAX (BioLegend, San Diego, CA, USA) following the manufacturer’s protocol. Tetramethylbenzidine colorimetric substrate was used to develop the blue color, and the optical density (450 nm) was measured using a microplate reader (Imark, Bio-Rad, Hercules, CA, USA).

### 4.7. Cell Death Detection ELISA

Necrosis was evaluated with photometric enzyme immunoassay to determine histone-associated DNA fragments (mono- and oligonucleosomes) (cell death detection ELISA, Roche Diagnostics GmbH, Mannheim, Germany) as previously reported [16]. Briefly, 96-well plates were coated with 100 μL of anti-histone antibody resuspended in coating solution (anti-histone antibody reacts with the histones H1, H2A, H2B, H3, and H4). The plate was incubated for 1 h at RT with gentle shaking. After removing the coating solution, the wells were rinsed three times with a washing solution. Next, the plate was incubated with 200 μL/well of coating buffer under the above-described conditions, and after, it was washed three times. Then, 100 μL of supernatant infection culture was diluted at 1:50 and added to each well, and the plate was incubated for 90 min at RT, with gentle shaking. After a wash cycle, 100 μL of conjugate anti-DNA-POD was added to each well (the anti-DNA-POD antibody binds to ss- and dsDNA). The plate was incubated for 90 min at RT, with gentle shaking. After a wash cycle, 100 μL of substrate solution (ABTS) was added. Finally, the absorbance was measured at 405 nm using 490 nm as the reference wavelength with a microplate reader (Imark, Bio-Rad, Hercules, CA, USA). Relative necrosis was calculated by normalizing OD values of infected conditions with uninfected conditions.

### 4.8. Statistical Analysis

Data are shown as mean ± standard deviation (SD). To compare more than two groups, the Kruskal–Wallis test was used, followed by Dunn’s post hoc test. Values of *p* < 0.05 were considered statistically significant (GraphPad Software, Inc., San Diego, CA, USA).

## 5. Conclusions

Our data provide evidence that the virulence of *M. tb* affects the cell death mechanisms that are activated in the macrophage after infection. It is well known that virulent *M. tb* develops multiple strategies to prevent apoptosis and favors necrosis to survive. However, this study shows that this could be a more complex phenomenon because the virulence of *M. tb* affects molecules of the BCL-2 family to block conventional apoptosis. Furthermore, at the same time, it alters the presence of molecules involved in other pathways that induce cell death, including apoptosis, necroptosis, and pyroptosis, suggesting that *M. tb* simultaneously alters these pathways. The crosstalk between pathways may be considered; altogether, they may be used as a strategy to ensure bacterial survival.

## Data Availability

The authors confirm that the raw data supporting the conclusions of this study are included in the manuscript. The corresponding author will provide more information, upon reasonable request, to any qualified researcher.

## Appendix A

**Table A1:** Antibodies used for flow cytometry and Western blot.

Antibody	Clone	Fluorochrome/ Catalog Number	Company
CD14	Monoclonal HCD14	FITC	BioLegend
CD2	Monoclonal TS1/8	BV421	BioLegend
CD19	Monoclonal HIB19	PE Cy7	BioLegend
BCL-2	Polyclonal	2872	Cell Signaling
Phospho-BCL-2 (Ser70)	Monoclonal, 5-H2	2827S	Cell Signaling
MCL-1	Polyclonal	4572s	Cell Signaling
BAK	Monoclonal, D2D3	6947s	Cell Signaling
Caspase-9	Monoclonal, 13D9C60	679101	BioLegend
Caspase-8	Monoclonal, 84131	MAB704	R&D
Caspase-3	Monoclonal, Poly6341	634101	BioLegend
ASK1	Polyclonal	AF3575	R&D
JNK1/2	Monoclonal, 252323	MAB2076	R&D
Phospho-RIP1 (Ser166)	Polyclonal	PA5-104645	Thermo Scientific
Phospho-RIP3 (Ser232)	Polyclonal	PA5-105701	Thermo Scientific
Phospho-p38 MAP Kinase (T180/Y182)	Polyclonal	AF869	R&D
BAX	Monoclonal, D2E11	5023S	Cell Signaling
DIABLO	Monoclonal, D5S3R	15108S	Cell Signaling
Β-ACTIN	Monoclonal, D6A8	8457	Cell Signaling
GAPDH	Monoclonal, D16H11	5174	Cell Signaling
Sheep	Polyclonal	HAF016	R&D
rabbit	Polyclonal	HAF008	R&D
mouse	Polyclonal	HAF007	R&D

## References

[1] World Health Organization (WHO) (2021). Global Tuberculosis Report. https://www.who.int/teams/global-tuberculosis-programme/tb-reports/global-tuberculosis-report-2021.

[2] Mertens C., Marques O., Horvat N.K., Simonetti M., Muckenthaler M.U., Jung M. (2021). The Macrophage Iron Signature in Health and Disease. Int. J. Mol. Sci..

[3] Shapouri-Moghaddam A., Mohammadian S., Vazini H., Taghadosi M., Esmaeili S.A., Mardani F., Seifi B., Mohammadi A., Afshari J.T., Sahebkar A. (2018). Macrophage Plasticity, Polarization, and Function in Health and Disease. J. Cell. Physiol..

[4] Orecchioni M., Ghosheh Y., Pramod A.B., Ley K. (2019). Macrophage Polarization: Different Gene Signatures in M1(Lps+) vs. Classically and M2(LPS-) vs. Alternatively Activated Macrophages. Front. Immunol..

[5] Beham A.W., Puellmann K., Laird R., Fuchs T., Streich R., Breysach C., Raddatz D., Oniga S., Peccerella T., Findeisen P. (2011). A TNF-Regulated Recombinatorial Macrophage Immune Receptor Implicated in Granuloma Formation in Tuberculosis. PLoS Pathog..

[6] Rodriguez-Cruz A., Vesin D., Ramon-Luing L., Zuñiga J., Quesniaux V.F.J., Ryffel B., Lascurain R., Garcia I., Chávez-Galán L. (2019). CD3^+^ Macrophages Deliver Proinflammatory Cytokines by a CD3- and Transmembrane TNF-Dependent Pathway and Are Increased at the BCG-Infection Site. Front. Immunol..

[7] Chavez-Galan L., Vesin D., Blaser G., Uysal H., Benmerzoug S., Rose S., Ryffel B., Quesniaux V.F.J., Garcia I. (2019). Myeloid Cell TNFR1 Signaling Dependent Liver Injury and Inflammation upon BCG Infection. Sci. Rep..

[8] Ramon-Luing L.A., Carranza C., Téllez-Navarrete N.A., Medina-Quero K., Gonzalez Y., Torres M., Chavez-Galan L. (2021). Mycobacterium Tuberculosis H37Rv Strain Increases the Frequency of CD3^+^TCR^+^ Macrophages and Affects Their Phenotype, but Not Their Migration Ability. Int. J. Mol. Sci..

[9] Moule M.G., Cirillo J.D. (2020). Mycobacterium Tuberculosis Dissemination Plays a Critical Role in Pathogenesis. Front. Cell. Infect. Microbiol..

[10] Mohareer K., Asalla S., Banerjee S. (2018). Cell Death at the Cross Roads of Host-Pathogen Interaction in Mycobacterium Tuberculosis Infection. Tuberculosis.

[11] Xu X., Lai Y., Hua Z.C. (2019). Apoptosis and Apoptotic Body: Disease Message and Therapeutic Target Potentials. Biosci. Rep..

[12] Warren C.F.A., Wong-Brown M.W., Bowden N.A. (2019). BCL-2 Family Isoforms in Apoptosis and Cancer. Cell Death Dis..

[13] Peña-Blanco A., García-Sáez A.J. (2018). Bax, Bak and beyond—Mitochondrial Performance in Apoptosis. FEBS J..

[14] Theobald S.J., Gräb J., Fritsch M., Suárez I., Eisfeld H.S., Winter S., Koch M., Hölscher C., Pasparakis M., Kashkar H. (2021). Gasdermin D Mediates Host Cell Death but Not Interleukin-1β Secretion in Mycobacterium Tuberculosis-Infected Macrophages. Cell Death Discov..

[15] Beckwith K.S., Beckwith M.S., Ullmann S., Sætra R.S., Kim H., Marstad A., Åsberg S.E., Strand T.A., Haug M., Niederweis M. (2020). Plasma Membrane Damage Causes NLRP3 Activation and Pyroptosis during Mycobacterium Tuberculosis Infection. Nat. Commun..

[16] Chávez-Galán L., Ramon-Luing L.A., Torre-Bouscoulet L., Pérez-Padilla R., Sada-Ovalle I. (2013). Pre-Exposure of Mycobacterium Tuberculosis-Infected Macrophages to Crystalline Silica Impairs Control of Bacterial Growth by Deregulating the Balance between Apoptosis and Necrosis. PLoS ONE.

[17] Afriyie-Asante A., Dabla A., Dagenais A., Berton S., Smyth R., Sun J. (2021). Mycobacterium Tuberculosis Exploits Focal Adhesion Kinase to Induce Necrotic Cell Death and Inhibit Reactive Oxygen Species Production. Front. Immunol..

[18] Zhao X., Khan N., Gan H., Tzelepis F., Nishimura T., Park S.Y., Divangahi M., Remold H.G. (2017). Bcl-XL Mediates RIPK3-Dependent Necrosis in M. Tuberculosis-Infected Macrophages. Mucosal Immunol..

[19] Tenev T., Bianchi K., Darding M., Broemer M., Langlais C., Wallberg F., Zachariou A., Lopez J., MacFarlane M., Cain K. (2011). The Ripoptosome, a Signaling Platform That Assembles in Response to Genotoxic Stress and Loss of IAPs. Mol. Cell.

[20] Pajuelo D., Gonzalez-Juarbe N., Tak U., Sun J., Orihuela C.J., Niederweis M. (2018). NAD+ Depletion Triggers Macrophage Necroptosis, a Cell Death Pathway Exploited by Mycobacterium Tuberculosis. Cell Rep..

[21] Davis R.J. (2000). Signal Transduction by the JNK Group of MAP Kinases. Cell.

[22] Yue J., López J.M. (2020). Understanding MAPK Signaling Pathways in Apoptosis. Int. J. Mol. Sci..

[23] Bergsbaken T., Fink S.L., Cookson B.T. (2009). Pyroptosis: Host Cell Death and Inflammation. Nat. Rev. Microbiol..

[24] Carranza C., Chavez-Galan L. (2019). Several Routes to the Same Destination: Inhibition of Phagosome-Lysosome Fusion by Mycobacterium Tuberculosis. Am. J. Med. Sci..

[25] Chai Q., Wang L., Liu C.H., Ge B. (2020). New Insights into the Evasion of Host Innate Immunity by Mycobacterium Tuberculosis. Cell. Mol. Immunol..

[26] Kim J.K., Silwal P., Jo E.K. (2020). Host-Pathogen Dialogues in Autophagy, Apoptosis, and Necrosis during Mycobacterial Infection. Immune Netw..

[27] Chávez-Galán L., Sada-Ovalle I., Baez-Saldaña R., Chávez R., Lascurain R. (2012). Monocytes from Tuberculosis Patients That Exhibit Cleaved Caspase 9 and Denaturalized Cytochrome c Are More Susceptible to Death Mediated by Toll-like Receptor 2. Immunology.

[28] Khan N., Downey J., Sanz J., Kaufmann E., Blankenhaus B., Pacis A., Pernet E., Ahmed E., Cardoso S., Nijnik A. (2020). *M. tuberculosis* Reprograms Hematopoietic Stem Cells to Limit Myelopoiesis and Impair Trained Immunity. Cell.

[29] de Zuani M., Frič J. (2022). Train the Trainer: Hematopoietic Stem Cell Control of Trained Immunity. Front. Immunol..

[30] Aguilo J.I., Alonso H., Uranga S., Marinova D., Arbués A., de Martino A., Anel A., Monzon M., Badiola J., Pardo J. (2013). ESX-1-Induced Apoptosis Is Involved in Cell-to-Cell Spread of Mycobacterium Tuberculosis. Cell. Microbiol..

[31] Arnett E., Schlesinger L.S. (2021). Live and Let Die: TB Control by Enhancing Apoptosis. Immunity.

[32] Dallenga T., Repnik U., Corleis B., Eich J., Reimer R., Griffiths G.W., Schaible U.E. (2017). *M. tuberculosis*-Induced Necrosis of Infected Neutrophils Promotes Bacterial Growth Following Phagocytosis by Macrophages. Cell Host Microbe.

[33] Lerner T.R., Borel S., Greenwood D.J., Repnik U., Russell M.R.G., Herbst S., Jones M.L., Collinson L.M., Griffiths G., Gutierrez M.G. (2017). Mycobacterium Tuberculosis Replicates within Necrotic Human Macrophages. J. Cell Biol..

[34] Rodel H.E., Ferreira I.A.T.M., Ziegler C.G.K., Ganga Y., Bernstein M., Hwa S.H., Nargan K., Lustig G., Kaplan G., Noursadeghi M. (2021). Aggregated Mycobacterium Tuberculosis Enhances the Inflammatory Response. Front. Microbiol..

[35] Dadsena S., Zollo C., García-Sáez A.J. (2021). Mechanisms of Mitochondrial Cell Death. Biochem. Soc. Trans..

[36] Divangahi M., Chen M., Gan H., Desjardins D., Hickman T.T., Lee D.M., Fortune S., Behar S.M., Remold H.G. (2009). Mycobacterium Tuberculosis Evades Macrophage Defenses by Inhibiting Plasma Membrane Repair. Nat. Immunol..

[37] Chen M., Divangahi M., Gan H., Shin D.S.J., Hong S., Lee D.M., Serhan C.N., Behar S.M., Remold H.G. (2008). Lipid Mediators in Innate Immunity against Tuberculosis: Opposing Roles of PGE2 and LXA4 in the Induction of Macrophage Death. J. Exp. Med..

[38] Chen M., Gan H., Remold H.G. (2006). A Mechanism of Virulence: Virulent Mycobacterium Tuberculosis Strain H37Rv, but Not Attenuated H37Ra, Causes Significant Mitochondrial Inner Membrane Disruption in Macrophages Leading to Necrosis. J Immunol.

[39] Sly L.M., Hingley-Wilson S.M., Reiner N.E., McMaster W.R. (2003). Survival of Mycobacterium Tuberculosis in Host Macrophages Involves Resistance to Apoptosis Dependent upon Induction of Antiapoptotic Bcl-2 Family Member Mcl-1. J. Immunol..

[40] Han L., Lu Y., Wang X., Zhang S., Wang Y., Wu F., Zhang W., Wang X., Zhang L. (2020). Regulatory Role and Mechanism of the Inhibition of the Mcl-1 Pathway during Apoptosis and Polarization of H37Rv-Infected Macrophages. Medicine.

[41] Adrain C., Creagh E.M., Martin S.J. (2001). Apoptosis-Associated Release of Smac/DIABLO from Mitochondria Requires Active Caspases and Is Blocked by Bcl-2. EMBO J..

[42] Pyrillou K., Burzynski L.C., Clarke M.C.H. (2020). Alternative Pathways of IL-1 Activation, and Its Role in Health and Disease. Front. Immunol..

[43] Man S.M., Tourlomousis P., Hopkins L., Monie T.P., Fitzgerald K.A., Bryant C.E. (2013). Salmonella Infection Induces Recruitment of Caspase-8 to the Inflammasome to Modulate IL-1β Production. J. Immunol..

[44] Weng D., Marty-Roix R., Ganesan S., Proulx M.K., Vladimer G.I., Kaiser W.J., Mocarski E.S., Pouliot K., Chan F.K.M., Kelliher M.A. (2014). Caspase-8 and RIP Kinases Regulate Bacteria-Induced Innate Immune Responses and Cell Death. Proc. Natl. Acad. Sci. USA.

[45] Lee J., Remold H.G., Ieong M.H., Kornfeld H. (2006). Macrophage Apoptosis in Response to High Intracellular Burden of Mycobacterium Tuberculosis Is Mediated by a Novel Caspase-Independent Pathway. J. Immunol..

[46] Obsilova V., Honzejkova K., Obsil T. (2021). Structural Insights Support Targeting ASK1 Kinase for Therapeutic Interventions. Int. J. Mol. Sci..

[47] Kishino A., Hayashi K., Maeda M., Jike T., Hidai C., Nomura Y., Oshima T. (2019). Caspase-8 Regulates Endoplasmic Reticulum Stress-Induced Necroptosis Independent of the Apoptosis Pathway in Auditory Cells. Int. J. Mol. Sci..

[48] Bertheloot D., Latz E., Franklin B.S. (2021). Necroptosis, Pyroptosis and Apoptosis: An Intricate Game of Cell Death. Cell. Mol. Immunol..

[49] Amaral E.P., Namasivayam S. (2021). Emerging Role for Ferroptosis in Infectious Diseases. Adv. Exp. Med. Biol..

[50] Halliwell B. (2020). Reflections of an Aging Free Radical. Free Radic. Biol. Med..

[51] Conrad M., Kagan V.E., Bayir H., Pagnussat G.C., Head B., Traber M.G., Stockwell B.R. (2018). Regulation of Lipid Peroxidation and Ferroptosis in Diverse Species. Genes Dev..

[52] Dodson M., Castro-Portuguez R., Zhang D.D. (2019). NRF2 Plays a Critical Role in Mitigating Lipid Peroxidation and Ferroptosis. Redox Biol..

[53] Haschka D., Hoffmann A., Weiss G. (2021). Iron in Immune Cell Function and Host Defense. Semin. Cell Dev. Biol..

[54] Amaral E.P., Costa D.L., Namasivayam S., Riteau N., Kamenyeva O., Mittereder L., Mayer-Barber K.D., Andrade B.B., Sher A. (2019). A Major Role for Ferroptosis in Mycobacterium Tuberculosis–Induced Cell Death and Tissue Necrosis. J. Exp. Med..

[55] Luo K., Stocker R., Britton W.J., Kikuchi K., Oehlers S.H. (2022). Haem Oxygenase Limits Mycobacterium Marinum Infection-Induced Detrimental Ferrostatin-Sensitive Cell Death in Zebrafish. FEBS J..

[56] Zhang Y., Chen X., Gueydan C., Han J. (2018). Plasma Membrane Changes during Programmed Cell Deaths. Cell Res..

[57] Carranza C., Juárez E., Torres M., Ellner J.J., Sada E., Schwander S.K. (2006). Mycobacterium Tuberculosis Growth Control by Lung Macrophages and CD8 Cells from Patient Contacts. Am. J. Respir. Crit. Care Med..

